# Multidisciplinary Approach to Synchronous Prostate and Rectal Cancer: Current Experience and Future Challenges

**DOI:** 10.14740/jocmr1796w

**Published:** 2014-03-31

**Authors:** Charalampos Seretis, Fotios Seretis, Nikolaos Liakos

**Affiliations:** a Department of Colorectal Surgery, Good Hope Hospital, Heart of England NHS Foundation Trust, Birmingham, UK; b Department of General Surgery, Medical School, University of Patras, Greece; c Department of Urology, St. Katharinen-Hospital GmbH, Frechen, Germany

**Keywords:** Cancer, Rectal, Prostate, Multidisciplinary, Decision, Surgery, Radiotherapy, Chemotherapy, Pelvis

## Abstract

The management of synchronous prostate and rectal cancer is a challeging task for the general surgeons and urologists, due to the complex anatomy of the pelvis and the sequential significant effects on the patient’s functional independency and quality of life. As both rectal and prostate cancers still remain leading causes of death in the male population, along with the increase of the average life expectancy, it is certain that synchronous prostate and rectal cancer will be a clinical scenario that the clinicians of the future will encounter more frequently. Our aim is to perform a comprehensive review on the management of this oncological entity, focusing on the significance of multidisciplinary approach which will enable the formation of an accurate strategy plan, having at all times the patient in the center of desicion-making.

## Introduction

Prostate and rectal cancers account for the most common pelvic malignancies in the male population and can present either synchronously or metachronously [1]. Without a doubt, prostate and rectal cancers are more likely to present metachronously, with prior radiotherapy for prostate cancer being the main proposed link [2]. Despite the significant advances in radiotherapy administration techniques, it is believed, although still of clinical controversy, that previous external beam radiotherapy for prostate cancer predisposes to the development of rectal cancer, as the rectum, most specifically the anterior rectum, receives a dose of the radiotherapy beam [3, 4]. The relevant interaction is more blur when it comes to prostate brachytherapy [5, 6]; however, it must be mentioned that the conflicting findings regarding the impact of prostate radiotherapy in future rectal cancer development could be explained by the fact that the mutagenic effects of radiotherapy are accumulative in nature and therefore studies with long-term follow-up are needed to establish any possible associations.

As the majority of men will develop at some age prostate neoplasias and taking into account the high prevalence of rectal cancer, despite the implementation of screening protocols and the advances in terms of primary prevention for both of these malignancies, it would be not endangered to claim that the scenario of synchronous prostate and rectal cancer will be increasingly frequent. Moreover, considering the anatomic proximity of the prostate and the rectum, which enables a locally advanced cancer to invade in tissue continuity the other, it is evident that the accurate diagnostic work-up and treatment strategy requires a multidisciplinary approach, in order to optimize the standards of the care delivered and maintain the patient’s quality of life. Herein, our aim is to provide a comprehensive review of the published literature regarding the management of the, not as infrequent as believed to be, challenging scenario of synchronous prostate and rectal cancer in a framework of a patient-centered multidisciplinary approach.

## Methods

We performed a comprehensive review of the published literature in PubMed database, using “prostate cancer”, “rectal cancer”, “synchronous”, “simultaneous” and their combinations as key phrases. Studies published in other than the English language were not included in our review. The review of the literature was performed independently by two of the contributing authors (CS and NL).

## Results

Our literature search yielded eight papers which were suitable to the purpose of our review [7-14]; three were case reports and the remaining five referred to case series, describing a total of 23 cases with synchronous prostate and rectal cancers. In a general overview, the vast majority of patients presented with symptoms suggestive of a colorectal lesion and in the sequential diagnostic work-up, the suspicion of a concurrent prostate malignancy occurred as a result of an abnormal digital rectal examination or elevated PSA titer, fact which led to further imaging of the pelvis and if appropriate the performance of biopsies of the prostate gland. However, in one of the included studies [12], the authors performed a prospective screening assessment for prostate cancer in a group of 20 patients who were scheduled to undergo an abdominoperineal resection for rectal cancer, detecting a rectal cancerous lesion in three out of the 20 cases.

In terms of the most preferred surgical intervention with curative intent, 8/23 patients underwent radical retropubic prostatectomy and abdominoperineal excision or low anterior resection depending on the tumor location, while 2/23 were submitted to pelvic exenteration with formation of ileal or colonic conduit respectively. Another option adopted was the utilization of chemoradiotherapy with or without androgen deprivation therapy aiming to achieve maximal regression primarily of the prostate cancer, followed by either low anterior or abdominoperineal resection for the remaining rectal tumor (4/23 cases).

The patients’ demographics, presenting features, histological characteristics of the prostate and rectal cancers, as well as the treatment strategies followed on each occasion and the outcome of the interventions were retrieved and are presented in a detailed manner in Tables 1 and 2.

**Table 1:** Presentation of Patients’ Demographics, Presenting Features, Histological Characteristics of the Prostate and Rectal Cancers, as Well as the Treatment Strategies Followed on Each Occasion and the Outcome of the Interventions

Authors	Age	Presenting feature(s)	Prostate cancer features	Rectal cancer features	Treatment	Postoperative complications	Outcome
Klee et al [7]	52	PSA 9.1 ng/mL	Gleason grade 7/10, stage T2C prostate adenocarcinoma right lateral wall	4 cm Duke’s stage B, moderately differentiated rectal adenocarcinoma	APR + radical rertropubic prostatectomy/blood loss 1,200 mL/operating time 4 h	Bowel obstruction and ischemic colostomy/LOS 16 days	At a minimum of 1 year of follow-up, all three patients had an undetectable PSA, and the rectal cancers remained in remission.
Klee et al [7]	70	PSA 5.9 ng/mL on routine rectal examination benign prostate, a left posterior lateral rectal wall mass	Gleason grade 6/10, stage T2A prostatic adenocarcinoma in right apex	2 cm Duke’s stage A, moderately differentiated rectal adenocarcinoma	APR + radical retropubic prostatectomy/blood loss 700 mL/operating time 3 h	Nil reported/LOS 8 days	
Klee et al [7]	64	PSA 6.1 ng/mL, change of bowel habits, induration in left prostate base and PR bleeding in DRE	Gleason grade 7/10 adenocarcinoma in left side and in the right base	4.5 cm non-invasive villous adenoma without cancerous features	LAR + radical retropubic prostatectomy/blood loss 1,100 mL/operating time 2.25 h	Rectal and bladder neck stricture/LOS 6 days	
Siu et al [8]	72	PSA 9 ng/mL	Gleason grade 6/10 prostate cancer	Not reported	Radiotherapy	Non applicable	In 2-year follow-up colonoscopy revealed no recurrence and PSA level was 0.7 ng/mL.
Siu et al [8]	73	Rectal tumor detected by DRE, PSA 7.9 ng/mL	Gleason grade 7/10 adenocarcinoma	3 cm T3 rectal cancer at dentate line	5-FU and radiotherapy	Non applicable	In 1-year follow-up no evidence of residual or recurrent rectal malignancy, PSA level 0.5 ng/mL.
Lin et al [9]	68	Rectal bleeding-rectal mass at DRE, PSA 57 ng/mL	T2N2M0 prostate adenocarcinoma (Gleason grade 5 (3 + 2))	3 cm T4N0M0 (Dukes’ stage C) rectal adenocarcinoma	LAR + radical retropubic prostatectomy + FOLFOX4, switched to FORFILI and then to capecitabine	Not reported	Death due to recurrence 47 months postoperatively
Lin et al [9]	65	Rectal bleeding-rectal mass at DRE, PSA 27 ng/mL	T3N0M0 prostate adenocarcinoma (Gleason grade 4 (2 + 2))	3 cm T4N1M0 (Dukes’ stage C) rectal adenocarcinoma	LAR + radical retropubic prostatectomy + FOLFOX4	Not reported	In 20-month follow-up asymptomatic
Lin et al [9]	70	Rectal bleeding-rectal mass at DRE, PSA 65 ng/mL	T3N1M0 prostate adenocarcinoma (Gleason grade 5 (2 + 3))	T3N0M0 (Dukes’ stage B) rectal adeno­carcinoma	APR + radical retropubic prostatectomy + FOLFOX4	Not reported	In 24-month follow-up asymptomatic
Ayhan et al [10]	84	Hematemesis and rectal mass detected by DRE	T2N0M0 prostate adenocarcinoma	T3N1M1 rectal adenocarcinoma	APR	Pulmonary edema + respiratory infection	Died immediate post-operative period

Abbreviations: PSA: prostate specific antigen; DRE: digital rectal examination; APR: abdominoperineal resection; LAR: low anterior resection; LOS: length of stay.

**Table 2:** Presentation of Patients’ Demographics, Presenting Features, Histological Characteristics of the Prostate and Rectal Cancers, as Well as the Treatment Strategies Followed on Each Occasion and the Outcome of the Interventions

Authors	Age	Presenting feature(s)	Prostate cancer features	Rectal cancer features	Treatment	Postoperative complications	Outcome
Ozsoy et al [11]	68	PSA 10 ng/mL	cT3aN0M0/Gleason grade 8	pT3N0 rectal adenocarcinoma	Radiotherapy	Non-applicable	Dead due to liver metastases from rectal primary/no evidence of prostate cancer recurrence at 1.08 years follow-up
Kavanagh et al [12]	n = 9 patients (mean age: 67.8 ± 10.3 years)	Rectal bleeding (n = 5), partial obstruction (n = 1), tenesmus (n = 1) and incidental during imaging for prostate cancer (n = 2)/elevated PSA in seven cases (mean PSA values: 21.4 ± 15.2 ng/mL)	Not reported	In group with no distant metastases (n = 5): ypT3N0, ypT4N1, ypT3N0, ypT3N1 (not reported for n = 1)	In group with no distant metastases (n = 5): 1) Radiotherapy + APR + prostatectomy (n = 1) 2) Radiotherapy + pelvic exenteration + ileal conduit (n = 1) 3) Radiotherapy + pelvic exenteration + colonic conduit (n = 1) 4) Radiotherapy + watchful waiting (n = 1) 5) Prostatectomy + CRT + LAR (n = 1) In group with distant metastases (n = 4): 1) Hormonal therapy + chemotherapy 2) Hormonal therapy + anterior resection 3) Hormonal therapy + palliation 4) watchful waiting/spinal decompression and radiotherapy	Wound infection (n = 2), foot drop + intra-abdominal collection (n = 1)/LOS: 33 ± 25.4 days	In group with no distant metastases (n = 5): dead with no evidence of recurrence after 10 years (n = 1), dead due to metastases after 29 months (n = 1), alive at 7 months with no evidence of recurrence (n = 1), alive at 4 years with metastases, alive at 3 months with no evidence of recurrence (n = 1) In group with distant metastases (n = 4): mean survival 8.5 months (range: 1 - 14 months)
Terris et al [13]	68	Not reported regarding rectal cancer/PSA 8.2 ng/mL	Stage T1c, Gleason grade 3 + 3 prostate cancer	Not reported	Preoperative radiotherapy + APR	Not reported	Alive at 15 months follow-up with PSA 0.5 ng/mL/No data regarding rectal cancer
Terris et al [13]	72	Not reported regarding rectal cancer/PSA 7.9 ng/mL	Stage T2a, Gleason grade 3 + 4 prostate cancer	Not reported	Preoperative radiotherapy + APR	Not reported	Alive at 10 months follow-up with PSA < 0.5 ng/mL/No data regarding rectal cancer
Terris et al [13]	73	Not reported regarding rectal cancer/PSA 32.4 ng/mL	Stage T3, Gleason grade 4 + 4 prostate adenocarcinoma	Not reported	Androgen deprivation therapy + APR	Not reported	Alive at 10 months follow-up with PSA 9 ng/mL/No data regarding rectal cancer
Colonias et al [14]	58	Rectal bleeding-rectal mass at DRE, PSA 32 ng/mL	stage II (T1cN0M0) prostate adenocarcinoma	stage III (T3N1M0), moderately differentiated rectal adenocarcinoma	Neoadjuvant CRT with androgen blockage, followed by proctosigmoidectomy and adjuvant chemotherapy with 5-FU and leucovorin	Not reported	In 14-month follow-up asymptomatic with PSA 0.3 ng/mL

Abbreviations: PSA: prostate specific antigen; DRE: digital rectal examination; APR: abdominoperineal resection; LAR: low anterior resection; LOS: length of stay.

## Conclusions

Despite being an uncommon co-incidental finding, the existence of synchronous prostate and rectal cancer will be a clinical problem that will be encountered more frequently due to the increase in life expectancy, since they still remain two of the most frequent malignancies in the male population. Taking into consideration the complexity of a surgical intervention with a curative intent and the high likelihood to perform extended resections without being able to preserve the autonomic function, it is essential that careful preoperative planning and thorough discussion with the patients is performed, explaining the balance between optimal oncological surgery and preservation of the functional anatomy with better the quality of life with tissue sparing surgery. Moreover, the existence of multiple options in terms of neoadjuvant and adjuvant therapies may enable effective local control at least of one of the two primary tumors and sequentially be followed by a more oncologically feasible operation.
